# Missed opportunities: are residents prepared to care for transgender patients? A study of family medicine, psychiatry, endocrinology, and urology residents

**Published:** 2018-07-27

**Authors:** Alexandre Coutin, Sarah Wright, Christine Li, Raymond Fung

**Affiliations:** 1 Faculty of Medicine, University of Toronto, Ontario, Canada; 2 Department of Medical Education, University of Toronto, Ontario, Canada; 3 Department of Family and Community Medicine, University of Toronto, Ontario, Canada; 4 Division of Endocrinology, Department of Medicine, Michael Garron Hospital, University of Toronto, Ontario, Canada

## Abstract

**Background:**

The transgender (trans) population faces multiple barriers in accessing health care, with knowledge deficits of health care providers contributing substantially. Trans patients report having to teach health care professionals about their own health needs. We compared perceptions of trans-care education and training across family medicine, psychiatry, endocrinology, and urology residency training programs at the University of Toronto.

**Methods:**

We surveyed residents to assess their perceptions of and attitudes towards trans-care, exposure to trans patients, knowledge of trans-specific clinical care, and the state of trans-care education within their training. We used Likert scale data to identify patterns across residency programs. We collected open-ended responses to further explain quantitative findings where appropriate.

**Results:**

Of 556 residents approached, 319 participated (response rate = 57.4%). Nearly all endocrinology and psychiatry residents agreed that trans-care falls within their scope of practice, while only 71% and 50% of family medicine and urology residents did, respectively. Though participants were at different stages of their postgraduate training when surveyed, only 17% of all participants predicted they would feel competent to provide specialty-specific trans-care by the end of their residency and only 12% felt that their training was adequate to care for this population.

**Conclusion:**

Though the study revealed a willingness to serve this population, there was a lack of clinical exposure and trans-related teaching within postgraduate curricula resulting in feelings of unpreparedness to meet the health care needs of this underserved population.

## Introduction

The prevalence of people self-identifying as transgender (trans) (see glossary) varies from 0.4% to 1.3% of the population and is similar for both birth-assigned males and females^1^ depending on definition and methodology used.^2^ One study concludes that 0.6% of the population would be a conservative estimate.^3–5^ Despite this substantial proportion, trans and other gender diverse populations are among the most underserved populations in health care.^1,6–8^ Trans patients encounter health care practitioners (HCPs) who lack knowledge, and these patients are at times denied care altogether, resulting in cases of unsafe surgical practices and self-prescribed hormone therapy.^9–12^ Moreover, the poor access to transition-related care has been implicated in the high rate of suicide and suicide attempts among trans Ontarians.^13^

Trans-care encompasses the general health needs that apply to the trans community like any other patient population, but also the more specific needs of trans patients in the clinical setting.^9,14^ While awareness is required to ensure the respect of patients’ gender identities (for example, the use of appropriate pronouns), HCPs must strike a balance so as to not overly focus on gender identity to the detriment of more general person-centered health needs.^14^ Trans-care frequently involves a more proactive advocacy on behalf of the HCP to aid the transitioning patient as they traverse the health-care system. Transitioning for many patients may involve hormone therapy, mental health care, gender affirming surgeries, and caring for the complications thereof.^15,16^ Particular consideration must also be given to cancer screening, fertility preservation, family planning, sexual health, substance use, mental health, risk of sexual assault, risk of violence, and socioeconomic factors.^9,17–21^ Thus, comprehensive trans-care ideally involves both generalists and specialists alike.

A lack of inclusion of trans-related curricula both at the undergraduate and postgraduate levels has been identified as one of the causes of the poor access to care.^22^ For example, only 30% of medical schools in the United States and Canada reported having gender transition-related content, while most did not cover trans health issues at all.^23^ Program-specific studies in urology and plastic surgery also showed little to no trans-related content.^24^ While an overwhelming majority of Canadian medical students agreed that trans issues were important, fewer than 10% felt sufficiently knowledgeable to address these issues.^14^ A study of physicians also revealed multi-factorial barriers to providing care to trans people, many of which related to knowledge gaps inherent in a system designed for cisgender (see glossary) patients.^9^ Furthermore, a qualitative study of physicians and trans patients asserted that even doctors who are well-intentioned, but do not know specifics about trans-care, contribute to difficulties in care access by referring patients to specialists who often have unacceptably long wait lists, due to the limited number of physicians knowledgeable about and skilled at trans-care.^25^ Given the high rate of suicide amongst those awaiting transition-related care,^26^ the authors called upon all generalist physicians to have basic training not only in diagnosing gender dysphoria but also in prescription and continued monitoring of hormone therapy.^25^

Initial attempts to address these knowledge gaps have shown some promising results. Medical learner exposure to lesbian, gay, bisexual, trans, queer (LGBTQ) patients and standardized patients in safe education environments resulted in more positive attitudes, improved knowledge, and confidence in trans-care. Including trans-related content in the curriculum has been shown to increase residents’ willingness to prescribe transition-related hormone therapy.^27^ Thus, increased curricular content and exposure to trans patients have been suggested as starting points for overcoming barriers to adequate care. However, there is no consensus on how this content should be included or which specialties should be targeted. Many interventions focus on LGBTQ health overall, overlooking the fact that trans patients have specific health needs that do not pertain to other LGBQ patients. A larger ongoing debate relates to how students should be taught about marginalized patient populations, and whether this teaching should include systemic political and social forces that generate inequities.^28^ It certainly behooves learners to strive to appreciate the previous and ongoing systemic factors, forms of violence, and misuses of power that impact the wellbeing of trans communities, for such inequities cannot be separated from the experience of trans patients.

Recent changes at the policy level seek to improve access and reduce obstacles to trans-care.^29–32^ Professional societies, like the Canadian Psychiatric Association, and the Endocrine Society have released position statements calling for the need to improve health care for this population.^19,33^ While these are major steps in improving access to care for the currently underserved trans patient population, the knowledge gap identified by others is likely to persist without changes to curricula. While studies have been conducted to identify trans content included in undergraduate medical programs, less is known about what content is provided at the postgraduate specialty level, and how this might affect whether specific specialties understand trans-care to be within their professional purview. To identify and explore the nature of education gaps across specialties and how they might influence resident perceptions of their responsibilities to learn about trans-care, we set out to explore the attitudes, knowledge, and training in trans-care among family medicine, psychiatry, endocrinology, and urology residents. The ultimate goal of this inquiry is to inform how education can be used to help improve health care for the trans community.

## Methods

### Participants

We invited 556 University of Toronto (UofT) residents across fifteen hospital sites and four specialties – endocrinology, urology, psychiatry, and family medicine – to participate in the survey. We aimed to be as inclusive as possible while balancing the need for informed opinions. Endocrinology residents were surveyed at the end of their PGY 4 or PGY 5 year (endocrinology consists of a two-year residency program in PGY4 and PGY5). Urology residents were surveyed at the end of their PGY1 through PGY5 years. We decided to include all urology residents due to the small number of residents in the program. We invited psychiatry residents from PGY 2 to 5. Lastly, both PGY1 and PGY 2 family medicine residents were surveyed, mostly at the end of their PGY1 and PGY2 years.

We obtained ethics approval from the University of Toronto Health Sciences Research Ethics Board. We used a cross-sectional design and employed both a paper and online survey.

### Survey development

We created a structured survey tailored for each specialty based on existing literature related to trans-care and medical education. We refined the initial survey based on feedback from key stakeholders, such as endocrinology and urology physicians, residents, and medical education scientists. Next, we asked three urology residents for their feedback, which was incorporated into the final version.

The final surveys consisted of 22 items, designed to explore residents’ perceptions of trans-care, including its relevance to their specialty, how much exposure they have had to trans patients, the state of trans-care education within their current training, and how all this affects their knowledge of and attitudes towards trans-care. The structure of each of the four surveys was the same, with the content altered only to make questions pertinent to each of the different specialties. For example, we asked (A) endocrinology and family medicine residents whether they believed by the end of their residency that they would be able to competently prescribe hormone therapy, (B) urology residents whether they would be able to perform sexual reassignment surgery (we used an older term so respondents may understand what we meant more easily), and (C) psychiatry residents whether they would be able to assess for and give counselling on gender dysphoria.

We collected demographic data such as postgraduate year of study, age, and gender identity. The majority of the survey consisted of Likert scale questions with options: Strongly Disagree, Disagree, Neutral, Agree, and Strongly Agree. Some questions were followed up with an open-ended question to gain further information beyond the Likert scale responses. Participants were instructed to provide responses that would only be representative of their current postgraduate program. For endocrinology trainees in particular, this would not include their prior internal medicine training.

### Procedure

We contacted postgraduate program directors to attain approval to invite their learners to participate in the study. The lead author (AC) attended compulsory teaching sessions of the different programs to administer the paper survey. Hospital site directors and administrative personnel coordinated times at which paper-based surveys could be administered during core teaching sessions. In order to ensure the inclusion of participants who could not attend these large group mandatory sessions, we provided an identical on-line version of each of the four specialty-specific surveys through email, with a link to the electronic survey using *Qualtrics* software. To prevent respondents from completing both the on-line and paper surveys, the first question we asked in our on-line survey was if they had already completed the written survey, and if so to stop. This strategy resulted in a total of 245 paper-based responses, and 74 on-line based responses.

### Analysis

We exported online survey responses from *Qualtrics Insight Platform* to *Microsoft Excel* and combined them with the paper-based responses already entered. We analyzed survey results separately by specialty program. We employed *SPSS Statistics* version 24.0 for Macintosh (IBM Corp., Armonk, NY) to perform Pearson Chi-square analyses to identify potential associations between demographic data and responses, with the significance level established as 0.05. In Chi-square tests resulting in 20% of the expected counts being less than 5, we used likelihood ratio as a substitute indicator of association, with the significance level also established as 0.05. We employed descriptive statistics where appropriate. We subjected open-ended responses to content analysis to reveal any recurrent themes or data that would further elucidate quantitative findings.

## Results

We collected 319 responses, eight from endocrinology (response rate 80%), 14 from urology (response rate 82%), 210 from family medicine (response rate 54%), and 94 from psychiatry (response rate 61%) (Table 1). The mean age of the respondents was 28 years, with higher proportions of female than male respondents, except in urology. No respondent identified themselves as trans or otherwise gender-variant.

**Table 1:** Demographics of respondents. Number of respondents for each specialty is subdivided into postgraduate year (PGY) 1-6, along with a fellow from the psychiatry program (1), and recent graduates (G). Response rates for each specialty are reported based on numbers of trainees at the time of sampling, provided by each training program.

	Specialty				Total

	Endocrinology	Urology	Family Medicine	Psychiatry	

**Number of Respondents**	8	14	210	87	319

**PGY1**		1	119		120
**PGY2**		4	85	32	121
**PGY3**		4		23	27
**PGY4**	5	4		16	25
**PGY5**	3	1		11	15
**G**			6	1	7
**PGY6**				3	3
**Fellow**				1	1

**Response rate (%)**	80.0	82.4	54.4	60.8	57.4
**Mean Age (SD)**	29.5 (1.8)	28.9 (0.95)	28.1 (3.1)	29.0 (2.5)	28.4 (2.9)
Gender* Female	5	6	132	60	203
**Male**	3	8	78	27	116

* Our question regarding gender included: male, female, transgender, other:_____. No respondent identified as transgender or other.

**Figure 1:**
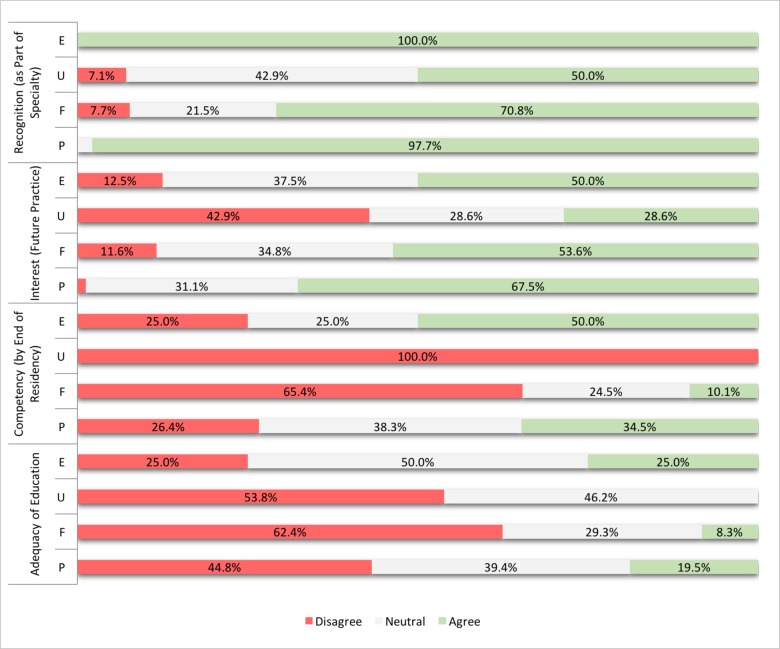
**Recognition, interest, and self-proclaimed competency and adequacy of education? with respect to transgender related care**. Participants in Endocrinology (E), Urology (U), Family Medicine (F), and Psychiatry (P) either strongly disagreed, disagreed, felt neutral about, agreed, or strongly agreed to each Likert scale statement, and responses were collapsed for more optimal interpretation. Residents were asked the extent to which they: believe transgender-related medical therapy should be within their scope of practice [Recognition]; would like to incorporate transgender care into their future practice [Interest]. Residents were also asked if by the end of their residency, they believe they will be able to competently prescribe hormone therapy (E,F), perform gender affirming surgery or deal with complications arising from such procedures (U), and competently assess for and give counseling on gender dysphoria (P) [Competency]. Lastly, they were asked whether they think teaching around treatment and management of the transgender population is adequate in their current curriculum [Adequacy of Education].

Recognition, interest, and self-reported competency regarding trans-care varied across these four disciplines (Figure 1). While an overwhelming majority of endocrinology (100%) and psychiatry (98%) residents recognized trans-related care to be part of their specialty, only 50% of urology and 71% of family practice residents felt this way. A substantial portion of psychiatry (68%), endocrinology (50%), and family medicine (54%) residents indicated having an interest in incorporating trans-related care in their future practice, whereas a lesser number of urology residents (29%) did. When respondents were prompted to comment about why they did or did not think trans-related therapy should be regarded as part of their specialty, they provided a diverse range of responses. Those who expressed positive views often cited wanting to be able to provide equitable care to all patients. Comments about interest and willingness to include trans-care into future clinical practice were recurrently paired with comments about lack of education, training, and exposure. Other respondents reported disinterest and beliefs that trans-care is too specialized or within the purview of another specialty’s care. One urology trainee notably responded “I am in surgery NOT psych!” to whether trans-related care should be regarded as part of urological care. Another suggested that physicians ought to be able to opt-out of treating this patient population. One participant cited religious beliefs as a reason for not wanting to treat this patient population; another two trainees stated trans-care is generally fraught with “too many ethical implications” and “too many issues.” Others cited difficulties and heightened complexity of care when treating this group as reasons not to participate. A frequently cited reason for not wishing to incorporate trans-care into future practice is not feeling comfortable handling this aspect of care. Several family medicine residents felt that trans-care was a “realm of specialized focus” and that there was “little [the resident physician] can do as a generalist to specifically serve this population, besides to be aware of/sensitive to their TG [transgender] state and provide usual core/referrals.”

While some endocrinology (50%) and psychiatry (35%) learners felt they would be competent at managing trans-related care in their respective roles by the end of their residency, very few family medicine (10%) and urology (0%) residents were similarly confident. In fact, very few residents in all disciplines thought that the current education around trans issues was adequate in their residency (Figure 1), keeping in mind that some residents surveyed were in their early years of their training.

**Figure 2:**
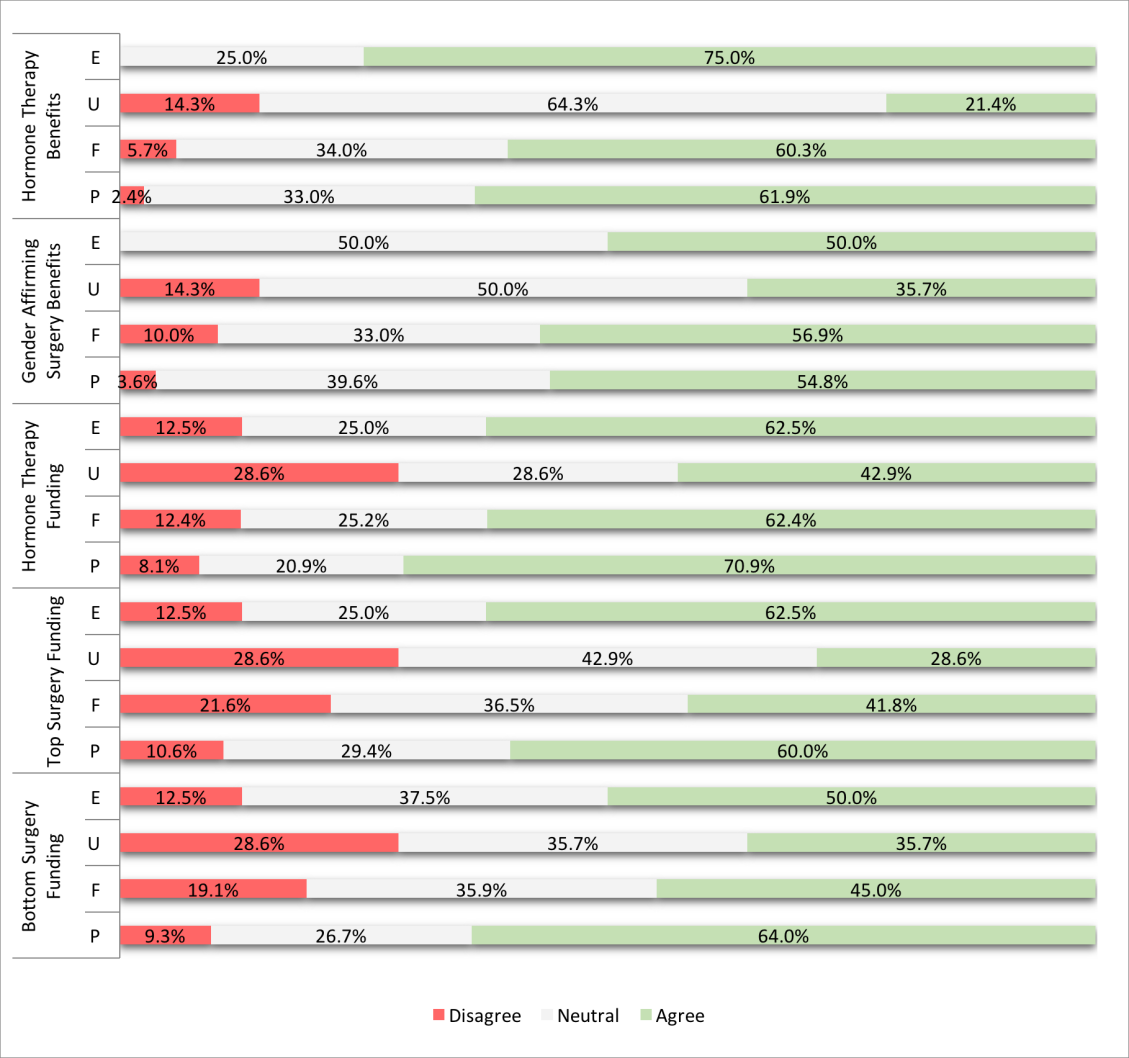
**Perception of and attitudes regarding transgender related clinical practice**. Residents were asked the extent to which they believe: the benefits of transgender hormone therapy outweigh its risks [Hormone Therapy Benefits]; the benefits of gender affirming surgery outweigh its risks [Gender Affirming Surgery Benefits]; hormone therapy should be fully government funded [Hormone Therapy Funding]; breast augmentation for trans women and chest contouring for trans men should be fully government funded [Tops Surgery Funding]; and gender affirming surgery should be fully government funded [Bottom Surgery Funding]. Endocrinology (E), Urology (U), Family Medicine (F), and Psychiatry (P).

With regards to attitudes and beliefs, a majority of residents felt that the benefits of hormone therapy and transition-related surgeries outweigh their risks, except for urology residents, who felt somewhat neutral about it (Figure 2). A similar pattern was found with regard to surgery. There were mixed feelings about whether hormone therapy or surgery should be fully funded by the government. Residents were more likely to believe that hormone therapy should be funded than surgical treatment, with urology residents least likely amongst the four groups to support funding for transition related therapy.

Most residents reported feeling comfortable when addressing general medical or surgical problems with transgender patients, with psychiatry residents being the most comfortable and willing to work with this population (Appendix A). Of particular interest, 21% of urology residents reported feeling uncomfortable seeing trans men for general urologic problems. Similarly, 16% of family medicine residents reported discomfort when seeing trans men or trans women for general medical issues. In contrast no endocrinology, and very few (<5%) psychiatry residents responded having this discomfort.

The majority (51%) of family medicine residents surveyed estimated that they had no hours of trans-related content in their postgraduate curriculum, while most (63%) of psychiatry residents had 1-3 hours. Many (43%) urology residents also reported having no hours of lecture/training, while endocrinology residents reported more (50% reported having 4-6 hours) (Appendix B). Endocrinology residents also reported having the most number of trans patient encounters (25% reported seeing more than 20 patients, 50% reported seeing 5-10 patients) while family medicine and urology reported having the fewest patient encounters (54% and 21% reported having no trans patient encounters, 41% and 57% having 1-5 encounters, respectively). Most psychiatry residents also only had 1-5 trans patient encounters (51%), but only 6.9% said they had no trans patient encounters. Residents expressed a desire to increase trans-related education content in multiple ways, with increasing trans patient exposure being the most popular choice; others wanted to have more experience prescribing and managing hormone therapy, increased lecture time as well as access to resources (Appendix C).

We did not uncover any statistically significant associations between demographic data and responses through our analysis.

## Discussion

This study revealed that residents in endocrinology, urology, psychiatry, and family practice are largely willing, yet feel unprepared, to care for trans patients. There is a gap between the proportion of residents who wish to incorporate trans-care into their future practice and those who feel they will be able to provide it upon graduation. This affirms that the current residency experience fails to provide adequate education to postgraduate trainees in four key specialties who otherwise could play a pivotal role in increasing access to care for trans patients. Residents who are willing to help, but who feel unprepared to provide this type of care, are likely to commence a chain of serial referrals for their patient, perpetuating the barriers to accessing timely and appropriate care that currently exist for trans individuals.^1,8^

Trainees in the two programs with more trans-care exposure (endocrinology and psychiatry) had the greatest self-reported competence, level of comfort, and recognition that trans-care fits within their specialty. Endocrinology residents reported more exposure to trans patients and curriculum content than others, and were the group that most frequently endorsed trans-care to be part of their specialty. Urology residents had the least curriculum exposure, and they were the least likely to have interest in providing trans-care, or even recognize it to be part of their specialty. It is therefore possible that an increase in curricular content would improve attitudes toward trans patient care, as is consistent with conclusions from previous studies.^27,34^ This study also suggests that in the specialty training programs in question, trans patient care is outside of the core curriculum. Given the intense workload during residency training, we cannot dependably rely on residents seeking their own experiences outside of the core curriculum. We must proactively include more formation, education, and training to eventually improve access to care for the trans population.

There were a number of striking results from family medicine respondents. Overwhelmingly they did not recognize trans-care as part of primary care; only half of family medicine residents cited interest in incorporating it as part of their future practice, and only 10% thought they would be competent enough to do so upon graduation. Primary care is the entry point for patients into the health care system. As such, family physicians play a vital role in helping trans patients attain the care they need and deserve. Indeed, past evidence has pointed to primary care practitioners not having the experience to be able to help their trans patients.^9,12,25^ However, recent guidelines aimed at improving access to care for trans patients indicate that hormone therapy is considered primary, not specialty, care.^35,36^ The increase in trans persons seeking care that accompanies a more progressive social landscape also raises the need for more HCPs proficient in prescribing and managing hormone therapy. Primary care is an appropriate space for this aspect of management, however our data suggest even budding family medicine graduates are willing yet unprepared to incorporate it.

There are many potential reasons for the lack of inclusion of trans-care education within these residency programs. First, faculty members themselves may lack expertise in this topic, and therefore, have been unable to teach and include it in their respective curricula. For example, there is currently no surgeon in Ontario performing vaginoplasty or phalloplasty surgeries, and therefore no opportunity to teach these procedures to residents. A survey of endocrinology programs in the United States found that a majority of faculty (80%) had never received training on caring for trans patients, and found that the lack of experience and knowledge amongst the faculty was a barrier to the provision of education for the residents.^37^ Second, the Royal College of Physicians and Surgeons of Canada, which sets the education standards of competencies residents must achieve, does not recognize trans-care as an important component of certain specialties. Management of “disorders of gender identity” is mentioned in the training objectives in Endocrinology and Metabolism, whereas no mention of trans-care is in the urology training objectives, which likely contributes to the experiences and attitudes of residents found in this study. Third, there may be a perception amongst practitioners and trainees that trans-care is only the responsibility of psychiatry, an outdated view that could change with further education about the neurobiological basis for gender variance. Indeed, this is reflected by a urology trainee’s empathic comment that they are “in surgery NOT psych!” amongst other participants’ comments about trans-care being bothersome, conflicting with religious beliefs, or unethical. Despite much progress towards an equitable landscape for trans individuals, it is a reality that these discriminatory and less-informed views endure in systems such as health-care. Intent aside, these assumptions decelerate the uptake of and dispel accountability for trans-care education. Practitioners often believe that endocrinology and psychiatry residents are responsible for trans-related care, and thus that residents in these specialties are receiving adequate education to meet the needs of trans patients. As this study has shown, that is not the case, pointing to a “someone else is taking care of it” phenomenon that leads to trans-care falling by the wayside. Future directions for research might involve qualitative inquiry into residents’ views of treating this patient population, or an expansion of the current study to other relevant specialties including pediatrics, emergency medicine, obstetrics and gynecology, and plastic surgery. This research may be critical in building consensus around competencies in relevant residency specialties around trans-care.

While this study clearly highlights a knowledge gap around trans patient care consistent with other studies, we are not convinced that the inclusion of curricular content and increased exposure to trans patients will solve the problem of inadequate trans-care. We agree with other authors who argue that simply viewing the fulfillment of a competency in the form of content acquisition is insufficient in ensuring future physicians are capable of addressing the care needs of a diverse society.^28,38^ They propose and we agree with developing a *critical consciousness* within our learners and orienting their armamentaria in a way that readies them for advocacy to counter social injustices and improve outcomes for communities at the margins of our health care system. This view contends that while the inclusion of curricular content around marginalized patient groups might be necessary, it is insufficient for making improvements to patient outcomes.^28^ We therefore advocate a dual approach to the problem, involving immediate curricular interventions to address the identified knowledge gap around trans patients, as well as a larger, longer-term transformative education approach in which learners are able to consider and engage with the social, cultural, and political conditions that have led to the marginalization of particular patient groups, including but not limited to trans patients.

Our study had a number of strengths. Trans-care involves multiple different specialties and we were able to survey a large number of residents across four different core specialties. We had favourable response rates from all four groups of residents, reassuring us that our data are likely representative of the views of these residents as a whole. Our method of distributing and collecting written surveys during mandatory academic sessions decreased the likelihood that only those residents most interested in the topic would participate in responding to the survey. We also have a number of limitations. As these are survey data, recall bias could have altered residents’ responses, and there is a limit to the accuracy of self-reported competence measures. We also included residents in earlier years of training which may have given us a less accurate reflection of the curriculum content as a whole. However, another key aspect of our study is the socialization that contributes to resident attitudes of what belongs in their specialty and what they feel their learning needs are. We feel this occurs early on in residency, and we therefore feel that these data are nevertheless valid and valuable. The study sample was limited to a single university. While the results of this study are largely descriptive, we feel that this exploratory approach was appropriate and has added to our knowledge about this understudied area of care.

### Conclusion

This study established a baseline of whether current postgraduate training programs in family medicine, endocrinology, urology, and psychiatry are providing learners with the tools to adequately care for the trans community. Our data suggest current residency training provides inadequate exposure and opportunity for education around trans-care. Residents feel unprepared to care for trans patients as they graduate from their training programs. However, a significant portion of residents desire more clinical exposure and training around trans-care, and wish to incorporate trans-care into their future practice. Taking steps to increase postgraduate learners’ exposure to the health care needs of trans patients while endeavoring to nurture a critical consciousness as to the importance of serving this vulnerable population will likely lead to improved care for the trans population.

## Appendix A

**Comfort level of providing general health care to transgender patients**. Residents were asked the extent to which they feel comfortable seeing: female-to-male transgender patients for general health problems related to their specialty [Comfortable Seeing Trans Men]; and male-to-female patients for general health problems related to their specialty [Comfortable Seeing Trans Women]. Endocrinology (E), Urology (U), Family Medicine (F), and Psychiatry (P).

## Appendix B

**Postgraduate curriculum**. Residents were asked how many encounters with transgender patients they have had in their residency training [Trans Patient Encounters Within Training] and how many hours of lecture/training they have received on transgender care in their residency curriculum [Hours of Training Within Curriculum].

## Appendix C

**Desired training improvements**. Residents were asked to select ways in which their training in transgender care could be improved by selecting all that apply from the following options: more lecture time, interaction with standardized transgender patients, case studies involving transgender patients, more exposure to transgender patients, more exposure to prescribing and monitoring hormone therapy, access to more transgender health care resources, and other. Respondents who selected “Other” were prompted to provide other potential improvements, however none of them did.

## Appendix D Glossary of terms

Bottom surgery: a type of gender affirming surgery (GAS), encompasses a variety of genital modification procedures. Typically vaginoplasty for trans women and metoidioplasty or phalloplasty for trans men.^35^

Cisgender: having a gender identity congruent with gender assigned at birth. Non-trans women are “cis women” and non-trans men are “cis men”.^35^

Gender identity : the internal and psychological sense of oneself as a woman, man, both, in between or neither. ^35^

Gender dysphoria: May refer specifically to the DSM-5 diagnosis and/or to the experience of distress associated with having their current gender presentation misaligned with their internal gender identity.^35^

LGBT/LGBTQ: acronyms used to refer to the lesbian, gay, bisexual, trans, and queer community. Certain acronyms may extend to include more sexual orientations or gender identities, while other acronyms may refer to a specific subset of the community being referred to in the literature (e.g., LGB is used to refer to cis persons who are lesbian, gay, or bisexual, without extending findings to trans persons).^35^

Metoidioplasty: gender affirming surgery, to create a penis and scrotum, done by cutting ligaments around the clitoris to add length to the shaft, grafting skin around the shaft to create added girth, lengthening the urethra, and creating a scrotum

Phalloplasty: gender affirming surgery, to create a penis and scrotal sac, and then to insert testicular and scrotal implants

Top surgery: chest modifying transition-related procedures. For trans men, this may involve removal of breast tissue and the contouring of a male chest. For trans women, this may involve breast augmentation.^35^

Transgender: having a non-cisgender identity; not identifying with one’s gender assigned at birth. Non-cis women are “trans women” and non-cis men are “trans men”.^35^

Trans: used interchangeably with transgender/ short for transgender

Transition: all changes involved in moving from one gender identity to another. This is a unique process for everyone who transitions, and may or may not include medical and/or surgical interventions.^35^

Trans man: previously referred to as a female-to-male (FTM) person, trans men are those assigned female at birth who transition to live on the masculine spectrum.^35^

Trans woman: previously referred to as a male-to-female (MTF) person, trans women are those assigned male at birth who transition to live on the feminine spectrum.^35^

Vaginoplasty: gender affirming surgery to create a vagina and vulva and remove the penis, scrotal sac and testes.
